# Obesity at age 20 and weight gain during adulthood increase risk of total and premature all-cause mortality: findings from women attending breast screening in Manchester

**DOI:** 10.1186/s12905-023-02162-0

**Published:** 2023-01-13

**Authors:** Mary Pegington, Michelle Harvie, Elaine F. Harkness, Adam Brentnall, Lee Malcomson, Jake Southworth, Jill Fox, Anthony Howell, Jack Cuzick, D. Gareth Evans

**Affiliations:** 1 The Prevent Breast Cancer Research Unit, The Nightingale Centre, Manchester University NHS Foundation Trust, Manchester, M23 9LT UK; 2 Division of Cancer Sciences, School of Medical Sciences, Faculty of Biology, Medicine and Health, The University of Manchester, Wilmslow Road, Manchester, M20 4BX UK; 3 Manchester Breast Centre, Manchester Cancer Research Centre, University of Manchester, 555 Wilmslow Rd, Manchester, M20 4GJ UK; 4 Division of Informatics, Imaging and Data Sciences, School of Health Sciences, Faculty of Biology, Medicine and Health, University of Manchester, Manchester, UK; 5 Centre for Cancer Prevention, Wolfson Institute of Preventive Medicine, Queen Mary University of London, London, UK; 6 Department of Medical Oncology, The Christie NHS Foundation Trust, Wilmslow Rd, Manchester, M20 4BX UK; 7 NW Genomic Laboratory Hub, Manchester Centre for Genomic Medicine, Manchester University Hospitals NHS Foundation Trust, Manchester, UK; 8 Genomic Medicine, Division of Evolution and Genomic Sciences, The University of Manchester, St Mary’s Hospital, Manchester University NHS Foundation Trust, Oxford Road, Manchester, M13 9WL UK

**Keywords:** Weight, Obesity, Women, Mortality, Premature mortality, Breast screening

## Abstract

**Background:**

Obesity in early adulthood is associated with lower breast cancer rates in later life. This could be interpreted as a positive reinforcement of excess weight amongst younger women however, the wider implications of higher weights are less well known. This study examined the association between both obesity in early adulthood and body mass index (BMI) change through adulthood, and all-cause mortality.

**Methods:**

The Predicting Risk of Cancer At Screening (PROCAS) study recruited 57,902 women aged 46–73 years (median age 57.2, IQR 51.8–63.7 years) from the Greater Manchester National Health Service breast screening programme in North West England between 2009 and 2015. It was used to assess associations between BMI at 20 years and cohort entry with all-cause mortality ascertained via deaths recorded on the National Breast Screening System to June 2020. Hazard ratios were estimated using proportional hazards (Cox) regression adjusted for factors at entry to the cohort: age, deprivation, bilateral oophorectomy, hormone-replacement therapy, menopausal status, ethnicity, alcohol intake, physical activity, and BMI.

**Results:**

The prevalence of overweight (25–30 kg/m^2^) and obesity (> 30 kg/m^2^) were 10.4% and 2.5% respectively at 20 years, increasing to 35.2% and 25.9% respectively at cohort entry. After a mean 8.7 years follow-up we observed that overweight (HR = 1.27, 95%CI = 1.10–1.47) and obesity (HR = 2.11, 95%CI = 1.67–2.66) at 20 years had a higher mortality rate compared with healthy weight. Women who were underweight/healthy weight at 20 years and gained weight to obesity at entry had a slightly increased mortality rate compared with women who were underweight/healthy weight at both time points (HR 1.16, 95%CI = 1.02–1.32). Women with overweight (HR = 1.36, 95%CI = 1.06–1.75) or obesity (HR = 1.90, 95%CI = 1.45–2.48) at both 20 years and entry had a higher mortality rate than women who were underweight/healthy weight at both points.

**Conclusions:**

Women who self-reported overweight and obesity at 20 years had a shorter life expectancy in this cohort of women attending breast cancer screening. Weight gain from 20 years was common in this group. Girls and women should be supported to maintain a healthy weight throughout the lifespan to help increase life expectancy.

*Trial registration number* NCT04359420, retrospectively registered 24/04/2020.

**Supplementary Information:**

The online version contains supplementary material available at 10.1186/s12905-023-02162-0.

## Background

Overweight (body mass index [BMI] 25–29.9 kg/m^2^), obesity (BMI ≥ 30 kg/m^2^) [1] and weight gain increase the risk of multiple diseases in women, including type 2 diabetes, cardiovascular disease (CVD), postmenopausal breast cancer (BC) and 12 other types of cancer [2–4]. These diseases all increase risk of mortality [5]. Rates of overweight and obesity for women in England have risen over the past 25 years; currently 60% are overweight including 29% with obesity [6]. BC is the most common cancer in women worldwide [7], and the second highest cause of cancer mortality in UK women [8]. Both the World Cancer Research Fund and the American Institute for Cancer Research state that weight gain during adulthood, and excess weight throughout adulthood increases risk of postmenopausal BC [7]. Conversely, excess weight between 18 and 30 years is associated with a reduced risk of both pre- and postmenopausal BC [7, 9].

One concern is that the potential benefits of early obesity on BC risk may be interpreted as a positive reinforcement of excess weight amongst younger women. Some studies have shown that higher weight in early adulthood is associated with an increase in all-cause mortality in women [10–12]. Using data from the Predicting Risk of Cancer At Screening (PROCAS) study, which recruited women from the NHS Breast Screening Programme in North West England, we have already shown the association of higher young adulthood weight with increased BC risk. We wished to look at the wider implications of early adulthood weight and weight change within this population of women attending breast screening in the UK. The primary aim of this study was to use data from the PROCAS cohort to assess all-cause mortality by BMI at 20 years and BMI change between 20 years and PROCAS entry. We hypothesized that increased BMI at 20 years, and increased weight gain during adulthood would be associated with increased all-cause mortality. Secondary aims were to assess premature mortality (defined by Public Health England as under 75 years [13]) by BMI at 20 years and BMI change to PROCAS entry, and compare causes of premature mortality.

## Methods

### Study design

The present analyses used a retrospective cohort study design using data from the PROCAS study. The design of the PROCAS study has been reported in detail elsewhere [14] and described in brief below.

### Population

In summary, between October 2009 and June 2015, 131,373 women aged 46–73 years in the Greater Manchester National Health Service (NHS) Breast Screening Programme in North West England were mailed PROCAS study information, consent form and a self-completed paper questionnaire including information on BC risk factors (Additional file 1: Fig. S1). Informed consent and completed questionnaires were obtained from 57,902 women (44%).

### BMI measurements and potential confounders

Weight at 20 years and PROCAS entry, and height were self-reported. BMI at 20 years and entry was categorised as underweight (< 18.5 kg/m^2^), healthy weight (18.5–24.9 kg/m^2^), overweight (25.0–29.9 kg/m^2^), and obese (≥ 30.0 kg/m^2^) [1].

Other variables from the questionnaire were included in the analysis if they were considered potential confounders for the relationship between BMI and all-cause mortality (age, deprivation score (English Indices of Multiple Deprivation [EIMD] 2010 derived from postcodes [15]), bilateral oophorectomy, hormone replacement therapy [HRT] use, menopausal status, ethnicity, weekly alcohol units and weekly physical activity [PA]).

### Data inclusion

**Fig. 1:**
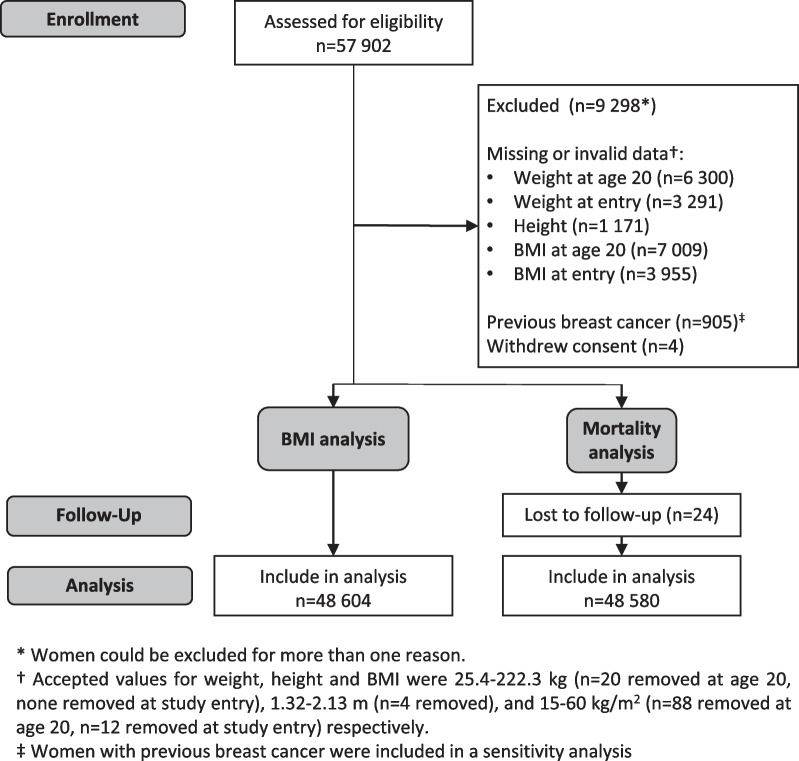
CONSORT 2010 flow diagram

Women with previous diagnosis of BC, or with unknown or invalid BMI at 20 years or PROCAS entry were excluded from the primary analyses (detailed in Fig. 1).

### Missing data and data cleaning

Women with missing menopausal status (n = 3 071) were considered post-menopausal if they were ≥ 55 years at entry (n = 370), otherwise they were considered pre/perimenopausal (n = 2 071 based on criteria by Phipps et al. [16]. Missing EIMD scores (n = 319) were imputed as the sample median of 18.35. Missing data on oophorectomy (n = 8869) and HRT (n = 538) were imputed as no bilateral oophorectomy and never taken HRT. Alcohol units and PA minutes per week were considered zero if the respondent answered no to drinking alcohol or no to moderate/vigorous activity in the last week respectively. If they had answered yes but had not entered the amount, sample medians were used (4.0 units and 210 min per week respectively).

### Death registration

The main outcome variable was time to death, and deaths were determined through the National Breast Screening System (NBSS, date of censoring: 15/06/2020). At date of censoring there were 1841 deaths of which 1546 (84%) were premature (< 75 years). Death certificates for premature deaths were requested through the General Register Office for all women with obesity at 20 years and known BMI at PROCAS entry, and no BC diagnosed before PROCAS entry (n = 81) and around 50 randomly selected deaths amongst women in two other weight change groups: (1) underweight/healthy weight at 20 years and healthy weight at PROCAS entry, (2) underweight/healthy weight at 20 years and obese at PROCAS entry. Two independent clinicians (AH and DGE) categorised the main cause of premature mortality as related to diabetes (type I and II), cardiovascular, cancers generally associated with obesity (oesophageal adenocarcinoma, gastric cardia, colorectum, liver, gallbladder, pancreas, post-menopausal breast, corpus uteri, ovary, renal cell, meningioma, thyroid, multiple myeloma [3]), other cancers or other causes.

### Statistical analysis

Summary statistics presented were sample mean and standard deviation (SD) if the distribution was approximately normally distributed, else median and interquartile range. Weekly self-reported alcohol intake and PA was compared with United Kingdom (UK) recommendations of maximum 14 units and minimum 150 min per week respectively. Proportional hazards (Cox) regression was used to estimate hazard ratios of BMI for all-cause mortality (Model 1 adjusted for age at study consent as continuous variable; Model 2 adjusted for categorical variables oophorectomy, HRT, menopausal status, ethnicity and continuous variables age at study consent, deprivation, alcohol units per week, PA minutes per week). The potential interaction between BMI at 20 years and PROCAS entry was tested using a likelihood-ratio test. Analysis was repeated for a premature mortality outcome, censoring women at date of last follow-up or age 75, whichever was earliest. Sensitivity analysis included women with previous BC (n = 905). Baseline characteristics of women with complete BMI data were compared to those with missing BMI at entry or age 20 years. In light of the high level of missing BMI data at 20 years (12%) and PROCAS entry (7%) we did a sensitivity analysis where they were included via multiple imputations (10 replicated), using Rubin’s rule for inference. Missing BMI was imputed using the Fully Conditional Specification Multiple Imputation method [17] using height, weight, hysterectomy, oophorectomy, menopausal status, HRT usage, PA, alcohol intake, alive status, and ethnicity. Cause of death was tabulated by BMI at 20 years and BMI change category, with healthy weight and underweight categories combined due to few deaths in these groups. Baseline characteristics of those included in the death certificate analyses were compared to those not included. Analysis used statistical software SPSS, version 25 (IBM, Armonk, New York, USA).

## Results

From 57 902 women entering the PROCAS study, 48,604 were eligible for inclusion in a complete cases analysis of weight and BMI, and 48,580 in the mortality analysis (Fig. 1). There were a total of 421,359 years of follow up to death or date of censor (mean follow-up 8.7 years).

### Baseline characteristics

Rates of overweight or obesity at 20 years were high amongst women from the most deprived decile, (overweight 18.3% and obesity 25.5%) compared with those from the least deprived decile (overweight 6.0% and obesity 3.6%) (Table 1). The majority of participants were white (95.6%), post-menopausal (67.6%), had never used HRT (62.8%) and 8.5% of the population had a bilateral oophorectomy. HRT usage was more likely amongst women who were underweight or a healthy weight at 20 years compared with women with obesity at 20 years (39.3% for underweight and 37.6% for healthy weight compared with 29.5% for obesity). Median reported alcohol intake was 4.0 (interquartile range [IQR] 0.0–10.0) units/week and 12.2% of women consumed more than the recommended maximum. A lower proportion of women with obesity at 20 years consumed alcohol above the recommended level and median intake was 1.0 unit/week (IQR 0.0–6.0) compared with 4.0 units/week (IQR 0.0–10.0) for underweight or healthy weight women. Median self-reported moderate intensity PA was 210 (IQR 60–480) min/week and 38.0% did not meet PA recommendations. Again, differences were observed between categories of BMI at 20 years with lower PA levels amongst women with obesity at 20 years, and a higher proportion not meeting PA recommendations (48.2% for obesity compared with 38.9 and 37.1% for underweight and healthy weight).

**Table 1:** Patient characteristics and health behaviours at PROCAS entry by BMI category at age 20 (n = 48 604)

	At age 20	Patient characteristics at PROCAS Study entry—by BMI category at age 20 (kg/m^2^)				
		All BMI categories	< 18.5	18.5–24.9	25.0–29.9	≥ 30.0
N (%)	48,604	48,604	3609 (7.4)	38,738 (79.7)	5082 (10.5)	1223 (2.5)
Age (years)^b^ Missing n = 0	–	57.2 (51.8–63.7)	57.1 (51.8–63.8)	57.4 (51.8–63.8)	56.8 (51.4–63.3)	56.7 (50.7–61.0)
Duration age 20 to PROCAS entry (years)^a^	–	38.0 (7.0)	37.9 (7.0)	38.1 (7.0)	37.7 (7.0)	36.2 (6.6)
Height (m)^a^ Missing n = 0	–	1.62 (0.07)	1.63 (0.07)	1.62 (0.06)	1.60 (0.07)	1.61 (0.07)
Weight at PROCAS entry (kg)^b^ Missing n = 0	57.2 (50.8–62.6)	68.9 (61.2–79.4)	62.6 (56.2–69.9)	68.0 (60.8–76.2)	82.1 (71.2–93.0)	95.4 (79.8–108.9)
BMI at PROCAS entry (kg/m^2^)^b^ Missing n = 0	21.6 (20.0–62.6)	26.3 (23.5–30.1)	23.4 (21.1–26.1)	26.0 (23.4–29.2)	31.8 (28.2–35.8)	37.1 (31.1–41.9)
BMI category at PROCAS entry^c^						
Underweight (< 18 kg/m^2^)	3609 (7.4)	376 (0.8)	169 (4.7)	204 (0.5)	3 (0.1)	0 (0.0)
Healthy weight (18–24.9 kg/m^2^)	38,738 (79.7)	18,523 (38.1)	2249 (62.3)	15,692 (40.5)	522 (10.4)	60 (4.9)
Overweight (25.0–29.9 kg/m^2^)	5034 (10.4)	17,104 (35.2)	873 (24.2)	14,739 (38.0)	1304 (25.9)	188 (15.4)
Obese (≥ 30.0 kg/m^2^)	1223 (2.5)	12,601 (25.9)	318 (8.8)	8103 (20.9)	3205 (63.7)	975 (79.7)
Missing n = 0						
English Index of Multiple Deprivation 2010 decile ^c^	–					
1 (most deprived)		6747 (14.0)	513 (14.3)	5009 (13.0)	915 (18.3)	310 (25.5)
2		4781 (9.9)	372 (10.4)	3645 (9.5)	585 (11.7)	179 (14.7)
3		4274 (8.8)	320 (8.9)	3320 (8.6)	5 498 (9.9)	136 (11.2)
4		4987 (10.3)	372 (10.4)	3941 (10.2)	543 (10.8)	131 (10.8)
5		4819 (9.9)	361 (10.0)	3874 (10.1)	472 (9.4)	112 (9.2)
6		4368 (9.0)	318 (8.9)	3519 (9.1)	441 (8.8)	90 (7.4)
7		4559 (9.4)	319 (8.9)	3736 (9.7)	436 (8.7)	68 (5.6)
8		4957 (10.2)	365 (10.2)	4033 (10.5)	467 (9.3)	92 (7.6)
9		4507 (9.3)	342 (9.5)	3762 (9.8)	349 (7.0)	54 (4.4)
10 (least deprived)		4340 (8.9)	311 (8.7)	3685 (9.6)	300 (6.0)	44 (3.6)
Missing		n = 265	n = 16	n = 214	n = 28	n = 7
Ethnicity ^c^	–					
White		47,141 (95.6)	3231 (92.5)	35,975 (95.8)	4692 (95.8)	1149 (96.5)
Asian or Asian British		613 (1.3)	126 (3.6)	436 (1.2)	40 (0.8)	11 (0.9)
Jewish		463 (1.0)	33 (0.9)	369 (1.0)	53 (1.1)	8 (0.7)
Black or Black British		351 (0.7)	36 (1.0)	262 (0.7)	42 (0.9)	11 (0.9)
Mixed		1 (< 0.1)	26 (0.7)	185 (0.5)	24 (0.5)	7 (0.6)
Other		425 (0.9)	41 (1.1)	331 (0.9)	48 (1.0)	5 (0.4)
Missing		n = 1 463	n = 116	n = 1 180	n = 135	n = 32
Menopausal status ^c^	–					
Pre/perimenopausal		15,737 (32.4)	1151 (31.9)	12,373 (31.9)	1700 (33.8)	513 (41.9)
Postmenopausal		32,867 (67.6)	2458 (68.1)	26,365 (68.1)	3336 (66.2)	710 (58.1)
Missing n = 0						
HRT (ever used)^c^	–	17,933 (37.2)	1420 (39.3)	14,469 (37.6)	1688 (33.8)	356 (29.5)
Missing		n = 370	n = 29	n = 277	n = 47	n = 17
Alcohol	–					
Units/week^b^		4.0 (0.0–10.0)	4.0 (0.0–10.0)	4.0 (0.0–10.0)	3.0 (0.0–9.0)	1.0 (0.0–6.0)
> 14 units/week^c^		5712 (12.2)	416 (12.0)	4709 (12.6)	514 (10.7)	73 (6.3)
Missing		n = 1 822	n = 143	n = 1 390	n = 223	n = 66
Physical activity						
Minutes/week^b^		210 (60–480)	210 (60–480)	240 (90–480)	180 (60–450)	150 (0–420)
PA < 150 min/week^c^		16,490 (38.0)	1244 (38.9)	12,865 (37.1)	1862 (41.6)	519 (48.2)
Missing	–	n = 5154	n = 415	n = 4030	n = 562	n = 147
Bilateral oophorectomy^c^	–	4109 (8.5)	306 (8.5)	3227 (8.3)	463 (9.2)	119 (9.7)
Missing		n = 7725	n = 562	n = 6191	n = 801	n = 171

^a^Mean (SD)

^b^Median (IQR 25th and 75th percentiles)

^c^n (% of valid data)

### BMI at 20 years and BMI changes

The majority of the population had a healthy BMI at 20 years (79.7%), compared with only 38.1% at PROCAS entry (mean [SD] 38.0 [7.0] years later). Overweight increased from 10.4 to 35.2%, and obesity increased from 2.5 to 25.9% (Table 1).

### BMI at 20 years and all-cause mortality rates

All-cause mortality rates were highest amongst those with obesity at 20 years (8.58 per 1 000 vs 4.07–5.59 for other BMI categories, Table 2). Regression analysis showed little evidence of an appreciable difference in HRs between underweight and healthy weight women at 20 years in any of the three models (Table 3 with full model fit in Additional file 2: Table S1). However, overweight and obesity at 20 years gave an increased HR for mortality compared with healthy weight in all models. In the fully adjusted model (including BMI at study entry) the HR for mortality was 1.27 (95%CI = 1.10–1.47) for overweight and 2.11 (1.67–2.66) for obesity compared with those who were a healthy weight at 20 years.

**Table 2:** Death rates by BMI category and BMI change category

	N	All deaths			Premature deaths		
		Length of follow up (person years)	Deaths (n)	Death rate per 1000 (95% CI)	Length of follow up (person years)^a^	Deaths (n)	Death rate per 1000 (95% CI)
BMI category age 20							
Underweight	3606	31,191.08	127	4.07 (3.41–4.83)	29,073.83	105	3.61 (2.70–4.35)
Healthy weight	38,720	336,317.58	1 382	4.11 (3.90–4.33)	312,590.58	1 158	3.71 (3.50–3.92)
Overweight	5032	43,476.08	243	5.59 (4.92–6.33)	40,525.17	202	4.99 (4.33–5.72)
Obese	1222	10,374.42	89	8.58 (6.93–10.51)	9744.00	81	8.31 (6.65–10.28)
BMI change category^b^							
Underweight or healthy weight → underweight or healthy weight	18,300	158,569.33	590	3.72 (3.43–4.03)	148,284.83	495	3.34 (3.05–3.64)
Underweight or healthy weight → overweight	15,607	135,874.42	544	4.00 (3.68–4.35)	125,596.33	451	3.59 (3.27–3.93)
Underweight or healthy weight → obese	8419	73,064.92	375	5.13 (4.63–5.67)	67,792.58	317	4.68 (4.18–5.21)
Overweight → underweight or healthy weight	525	4554.33	34	7.47 (5.25–10.31)	4200.92	30	7.14 (4.91–10.07)
Overweight → overweight	1304	11,287.67	68	6.02 (4.72–7.59)	10,347.25	51	4.93 (3.71–6.43)
Overweight → obese	3203	27,634.08	141	5.10 (4.31–6.00)	25,967.67	121	4.66 (3.88–5.55)
Obese → underweight or healthy weight	60	522.67	13	24.87 (13.83–41.46)	440.92	11	24.95 (13.12–43.36)
Obese → overweight	187	1596.33	16	10.02 (5.93–15.93)	1495.33	15	10.03 (0.58–1.62)
Obese → obese	975	8255.42	60	7.27 (5.60–9.29)	7807.75	55	7.04 (5.36–9.10)
Overall group	48,580	421,359.17	1841	4.37 (4.17–4.57)	391,933.58	1546	3.95 (3.75–4.15)

^a^From PROCAS entry to earliest of death, age if < 75 at time of censor, or age 75

^b^Due to low numbers of women underweight at entry and the focus of the analyses on obesity, the underweight and healthy weight categories were combined

**Table 3:** Association of BMI at age 20 with all-cause mortality (all ages)

BMI category at age 20	Number	Number of deaths (%)	Model 1		Model 2	
			A HR (95% CI)	B HR (95% CI)	A HR (95% CI)	B HR (95% CI)
Underweight	3606	127 (3.5)	1.01 (0.84–1.21)	1.06 (0.88–1.27)	1.00 (0.83–1.20)	1.01 (0.84–1.21)
Healthy weight	38,720	1382 (3.6)	1 (reference)	1 (reference)	1 (reference)	1 (reference)
Overweight	5032	243 (4.8)	1.41 (1.23–1.62)	1.28 (1.11–1.48)	1.30 (1.14–1.49)	1.27 (1.10–1.47)
Obese	1222	89 (7.3)	2.70 (2.17–3.34)	2.25 (1.78–2.84)	2.21 (1.78–2.74)	2.11 (1.67–2.66)

Cox regressions: Model 1 adjusted for age at consent; Model 2 adjusted for age at consent, deprivation level, bilateral oophorectomy, HRT, menopausal status, ethnicity, alcohol units per week, physical activity minutes per week. A: not adjusted for BMI at PROCAS entry; B: adjusted for BMI at PROCAS entry.

### BMI change and all-cause mortality rate

All-cause mortality rates were lowest amongst women underweight or healthy weight at both 20 years and PROCAS entry (3.72 per 1000) (Table 2). Regression analysis with full adjustment (Model 2) showed lowest mortality risks were seen amongst women who maintained at under/healthy weight or increased to overweight by PROCAS entry (Table 4 with full model fit in Additional file 3: Table S2), with evidence of an interaction between BMI at 20 years and entry (Additional file 3: Table S2). Compared with women underweight/healthy weight at both time points, women who were underweight/healthy weight at 20 years and gained weight to be in the obese category at PROCAS entry had a HR for mortality of 1.16 (95%CI = 1.02–1.32) in the fully adjusted model. Women maintaining at overweight or obese had increased mortality risks of 1.36 (1.06–1.75) and 1.90 (95%CI = 1.45–2.48) respectively. The three weight loss groups had relatively high hazard ratios for mortality though there were lower numbers of women in these groups thus wide confidence intervals.

**Table 4:** Association of BMI change between age 20 and joining PROCAS with all-cause mortality (all ages)

BMI category age 20	BMI category at PROCAS entry	Number	Number of deaths (%)	Model 1	Model 2
				HR (95% CI)	HR (95% CI)
Underweight or healthy weight	Underweight or healthy weight	18,300	590 (3.2)	1 (reference)	1 (reference)
	Overweight	15,607	544 (3.5)	1.01 (0.90–1.14)	0.94 (0.83–1.05)
	Obese	8419	375 (4.5)	1.38 (1.21–1.57)	1.16 (1.02–1.32)
Overweight	Underweight or healthy weight	525	34 (6.5)	1.89 (1.34–2.68)	1.76 (1.25–2.49)
	Overweight	1304	68 (5.2)	1.47 (1.15–1.89)	1.36 (1.06–1.75)
	Obese	3203	141 (4.4)	1.47 (1.23–1.77)	1.22 (1.02–1.47)
Obese	Underweight or healthy weight	60	13 (21.7)	6.74 (3.89–11.67)	5.05 (2.91–8.76)
	Overweight	187	16 (8.6)	3.45 (2.10–5.67)	2.83 (1.72–4.66)
	Obese	975	60 (6.2)	2.49 (1.91–3.25)	1.90 (1.45–2.48)

Cox regressions: Model 1 adjusted for age at consent; Model 2 adjusted for age at consent, deprivation level, bilateral oophorectomy, HRT, menopausal status, ethnicity, alcohol units per week, physical activity minutes per week. Reference categories: underweight or healthy weight at both time points, pre/perimenopausal, white ethnicity.

Due to low numbers of women underweight at entry and the focus of the analyses on obesity, the underweight and healthy weight categories were combined.

### Secondary analyses

#### Multiple imputation

Results from the regressions of all-cause mortality at any age using the imputation databases were similar to the primary analyses (Additional files 4 and 5: Tables S3 and S4).

#### Comparison of cases in included cohort with those with missing BMI at one or both time points

This revealed higher mortality and premature mortality rates amongst women not reporting BMI at one or both time points (Additional file 6: Table S5). Those excluded were younger at consent and from areas of higher deprivation.

#### BMI at 20 years, BMI change and premature mortality (< 75 years)

Premature mortality rates followed a similar pattern to those for total mortality (Table 2). Findings for the association of BMI 20 years and BMI change with premature mortality were similar but weaker results to those for all-cause mortality except that maintaining at overweight no longer gave a significantly increased HR (Additional files 7 and 8: Tables S6 and S7).

#### Inclusion of women with previous BC

Sensitivity analysis was performed on the above models including women diagnosed with BC before PROCAS entry with similar results to the previous analyses (Additional files 9, 10, 11, 12: Tables S8, S9, S10, S11).

#### Causes of premature mortality

Death certificates were obtained for 67/81 (82.7%) premature deaths amongst women with obesity at 20 years, the remainder were not available at the time of request. This analysis also included 49 of those underweight/healthy weight at 20 years and healthy weight at PROCAS entry, and 50 of those underweight/healthy weight at 20 years and with obesity at PROCAS. Therefore the analysis included 166/1546 (10.7%) of overall premature deaths. Comparison of characteristics of those included/not included in each of the three selection groups is shown in Additional file 13: Table S12. The groups were comparable in terms of age, deprivation, ethnicity, menopausal status and other key characteristics.

#### BMI at 20 years and cause of premature mortality amongst women with no previous history of BC

Amongst women with obesity at 20 years, the main causes of death were related to cancer (31.3%, of which 52.4% were cancers generally associated with obesity), CVD (26.9%), and other causes (35.8%, e.g. lung, liver or motor neurone disease) (Additional file 14: Table S13).

#### BMI change and cause of premature mortality amongst women with no previous history of BC

Deaths related to diabetes and cardiovascular conditions were highest in women with obesity at 20 years, entry, or both (diabetes all ≥ 4.5%, cardiovascular all ≥ 10.0% compared to being underweight/healthy weight at both time points: diabetes 0.0%, cardiovascular 8.2%) (Additional file 15: Table S14). In light of the analyses within this paper finding that women with obesity at 20 years are more likely to die prematurely than healthy weight women, this analysis of death certificates suggested that this is partly due to deaths from diabetes and cardiovascular reasons which were at lower levels in women with underweight/healthy weight at 20 years.

## Discussion

### Overall findings

This analysis highlighted a number of issues pertinent to women’s health; (1) many women gained significant amounts of weight between 20 years and middle age; (2) overweight and obesity at 20 years increased risk of overall and premature mortality even after adjustment for BMI in later adulthood; and (3) weight gain in adulthood after being underweight/healthy weight at 20 years also increased overall and premature mortality.

Our finding that BMI in young adulthood is positively correlated with all-cause mortality and the effect is independent of later BMI mirrors that of other large cohort studies that have adjusted for later BMI [10–12].

Our analyses suggest that women who are underweight/healthy weight at 20 years should remain in that category or increase at most to overweight, and our results concur with other large cohorts from America and Australia [10, 18–21].

The increased all-cause mortality risk seen here amongst the minority of women (1.6%) who moved to a lower BMI category is likely due to reverse causality, whereby a proportion of deaths are likely to be due to medical conditions which also resulted in weight loss preceding death as previously reported [22].

The present analysis indicated that the higher rates of premature mortality amongst women with obesity at 20 years and those experiencing an upward shift in BMI category from 20 years to mid-late adulthood are primarily driven by diabetes and CVD. Other studies have reported similar findings with respect to CVD [11, 19, 23], while other studies have shown a higher mortality rate from obesity-related cancers amongst these groups [24, 25].

### Strengths, limitations, and public health implications

The strengths of this study include the large sample size with a broad spread of deprivation scores and data on a number of confounders, minimal missing data and 421,359 women years of complete follow up data. We employed multiple imputation to reduce bias caused by missing data, and sensitivity analyses gave consistent results.

Our study encountered several limitations that warrant discussion. Firstly, there are limitations on the generalisability of the present results to the UK population. The PROCAS study recruited only 4.4% non-white participants when 9.8% of the population in North West England were non-white according to the 2011 census, though a lower proportion within the 46–73 year-old age group will be non-white as the non-white population of England and Wales has a lower average age [26]. Despite the broad spread of deprivation scores, the PROCAS cohort was less deprived than the general Greater Manchester population [27], a common issue in health research [28]. Thirty-eight percent of PROCAS participants had a healthy BMI compared with only 21.9% of women age 55–64 years in the Health Survey for England in 2012, the middle year of recruitment to PROCAS [29]. The lower rate of obesity in PROCAS compared to the Health Survey for England suggests that participants were more health conscious than the overall population and/or self-reported heights and weights were over/underestimated. It is recognised that women with overweight and obesity are more likely to both underestimate their weight, and overestimate their height [30]. However, assessing BMI at 20 years in this cohort by using recalled weight has previously been found to be reliable so this should have had minimal effect on the study outcomes [9]. Self-report is also likely to have resulted in underreporting of alcohol and PA data thus it is likely that fewer women adhered to recommendations than reported here. Women in this study were born before 1970 when obesity at 20 years was uncommon. Only 2.5% of the PROCAS sample had obesity at 20 years compared to 37% of current 16–24 year-old females in England [6]. Thus, analyses including this group had wide confidence intervals. Our findings indicate that women of breast screening age with obesity at 20 years are more likely to die prematurely than those with BMI < 25 kg/m^2^ at 20 years. However, we also note there is potential selection bias such as having fewer women with obesity at 20 years recruited to PROCAS as they were already unwell, or had died before they were recruited at age 46–73 years. In addition to the limitations on generalisability to the UK population, the present results may not be relevant to lower income countries.

Secondly, there are limitations concerning the robustness of the data collection. We excluded 8 385 women from the analyses due to missing BMI because height and/or weight were not completed on the questionnaire. The higher mortality rate amongst women not included in the analysis due to missing BMI could have been caused by a higher proportion of overweight/obesity amongst those not included, or due to the higher deprivation amongst women not included (which could have caused higher mortality directly, or indirectly via higher overweight/obesity levels). Therefore, the associations between weight gain and maintaining overweight/obesity seen here are likely to be stronger if all cases had been included. The analysis was based on self-reported weight at two time points and does not contain any data for weight changes in the intervening years. We also lack information on weight changes between PROCAS entry and the censor date of June 2020. More frequent objective measurement of weight would help determine whether total years spent with overweight/obesity should be included in mortality risk calculation models in a similar manner to the cumulative damage done by cigarette smoking and the concept of “pack years”. This would also help clarify the importance of age at weight gain/loss as some studies suggest that weight gain in early adulthood is more harmful than in later life [10, 19], whereas weight loss has been shown to be detrimental to mortality if it occurs in later life [19]. The questionnaire only collected weight data at age 20 years therefore no comment can be made on other changes between age 20 years and PROCAS entry such as change in alcohol intake or PA levels. Other confounders such as education level, marital status, dietary pattern, and other health problems could mediate the relationship between weight change and all-cause mortality. We did not collect this data thus could not adjust for these factors. In addition, we lack smoking data. Smoking is a major confounder as it is associated with lower weight, whilst increasing risk of mortality [22]. Twenty percent of women in North West England in 2011 were current smokers [31] so this could have impacted on the results by increasing the mortality rate amongst women in the lower weight categories. Deaths were determined through NBSS which is updated for women on the PROCAS study unless they transfer into another area’s NHS breast screening programme. Data on the proportion of 50–70 year-old women in Manchester and Trafford that move out of area annually suggest that up to 18% of this population could have left during the follow-up period so data on missing deaths could be significant [32, 33]. The cause of death analysis using death certificates was limited to a small number of available death certificates due to cost. This analysis was also limited by incomplete and potentially inaccurate reporting of cause of death. Unfortunately, several of those in our study did not follow UK guidelines [34] leading to inaccuracies in the classifications used here. For example, five cases were listed as metastatic carcinoma with no primary cause listed thus were classed as cancer deaths whereas they may have been cancers generally associated with obesity. Obesity is largely associated with type 2 but not type 1 diabetes. However, the type of diabetes was only specified in 4/5 cases, so we could not separate out weight-related cases. We have not distinguished between pre and post-menopausal BC. Only post-menopausal BC is associated with obesity thus some premenopausal BC cases in our analyses could be incorrectly classed as obesity-related cancers.

Thirdly, there are limitations concerning the analysis techniques. We have used multiple imputation to deal with missing data however this relies on data being missing at random, whereas there may be a bias to missing weight data amongst women with higher weights. We did not attempt to minimise impact of reverse causation unlike other studies which have excluded deaths within the first few years of follow-up (e.g. [24]).

Finally, it is important to note that the PROCAS study is still in follow up and the mortality figures presented here are not final, with 81% of the cohort < 75 years and still alive at the time of last follow-up hence the majority of deaths so far recorded are premature. Future research could repeat the current analysis when more of the cohort are deceased, or perform other analyses such as population attributable fraction which would estimate the burden of excess deaths related to overweight and obesity at age 20, and weight gain during adulthood.

Whilst analyses of the PROCAS cohort has previously shown that being overweight or obese at 20 years reduces risk of later BC [9], the current results together with those from other cohorts indicate higher weight in young adulthood significantly increases overall and premature mortality rates. Hence any potential benefits of obesity in early adulthood must not be overstated. With obesity rates amongst 13–15 year-old girls in the UK now at 18%, this is likely to have a substantial impact on health and healthcare costs in future years [6]. More resources should be channelled into helping children and adolescents avoid weight gain in order to prevent having overweight or obesity by 20 years. Results from this and other cohorts also highlight the benefit of avoiding weight gain during adulthood.

The optimal methods for supporting children, adolescents and adults to avoid weight gain are currently unknown. Future research could design and evaluate interventions with the overall aim of finding effective interventions that could be rolled out. Existing, peer-reviewed frameworks should be used such as guidance from the Medical Research Council on developing complex interventions [35] which includes development or identification of an intervention, assessment of feasibility of the intervention and evaluation design, evaluation of the intervention, and impactful implementation.

## Conclusion

We have shown in this large, UK cohort, that obesity at age 20 and weight gain through adulthood both increased overall and premature all-cause mortality risk. This study adds to the literature highlighting that present-day high levels of obesity in childhood, adolescence and early adulthood, as well as weight gain during adulthood may be detrimental to health in later life and reverse the trend in increased life expectancy. Action must be taken to help girls, female adolescents and young adults to avoid weight gain through the lifespan and future research could design and test effective interventions to achieve this.

## Supplementary Information

**Additional file 1. Figure S1:** PROCAS questionnaire.

**Additional file 2. Table S1:** Full list of coefficients for Table 3: Association of BMI at age 20 with all-cause mortality (all ages).

**Additional file 3. Table S2:** Full list of coefficients for Table 4: Association of BMI change between age 20 and joining PROCAS with all-cause mortality (all ages).

**Additional file 4. Table S3:** Table 3 with addition of multiple imputation: Association of BMI at age 20 with all-cause mortality (all ages).

**Additional file 5. Table S4:** Table 4 with addition of multiple imputation: Association of BMI change between age 20 and joining PROCAS with all-cause mortality (all ages).

**Additional file 6. Table S5:** Comparison of characteristics and total / premature death rates by those included / excluded in the cohort (due to missing BMI at one or both time points).

**Additional file 7. Table S6:** Association of BMI at age 20 with premature mortality (<75 years).

**Additional file 8. Table S7:** Association of BMI change between age 20 and joining PROCAS with premature mortality.

**Additional file 9. Table S8:** Sensitivity analysis including those with previous BC (n=49 374) - Association of BMI at age 20 with all-cause mortality (all ages).

**Additional file 10. Table S9:** Sensitivity analysis including those with previous BC (n=49 374) - Association of BMI change between age 20 and joining PROCAS with all-cause mortality (all ages).

**Additional file 11. Table S10:** Sensitivity analysis including those with previous BC - Association of BMI at age 20 with premature mortality.

**Additional file 12. Table S11:** Sensitivity analysis including those with previous BC - Association of BMI change between age 20 and joining PROCAS with premature mortality.

**Additional file 13. Table S12:** Characteristics of women who died prematurely included (n=166) and not included (n=1 380) in the cause of death analysis.

**Additional file 14. Tables S13 and S14:** Table 13: Main cause of premature death by BMI category at age 20, for a subset of premature deaths, n=166 and Table 14: Main cause of premature death by BMI change category, for a subset of premature deaths, n=166

## Abbreviations

BMI: Body mass index
CVD: Cardiovascular disease
EIMD: English Indices of Multiple Deprivation
HR: Hazard ratio
HRT: Hormone replacement therapy
IQR: Interquartile range
NBSS: National Breast Screening System
PA: Physical activity
PROCAS: Predicting Risk of Cancer at Screening
SD: Standard deviation
UK: United Kingdom
