# Supplementary figures and images for: Obesity at age 20 and weight gain during adulthood increase risk of total and premature all-cause mortality: findings from women attending breast screening in Manchester
Source: BMC Womens Health. 2023 Jan 13;23:17. doi: 10.1186/s12905-023-02162-0 (PMC9837983; doi:10.1186/s12905-023-02162-0)

**Additional File 1**

**Supplementary Figure 1: PROCAS questionnaire**

**
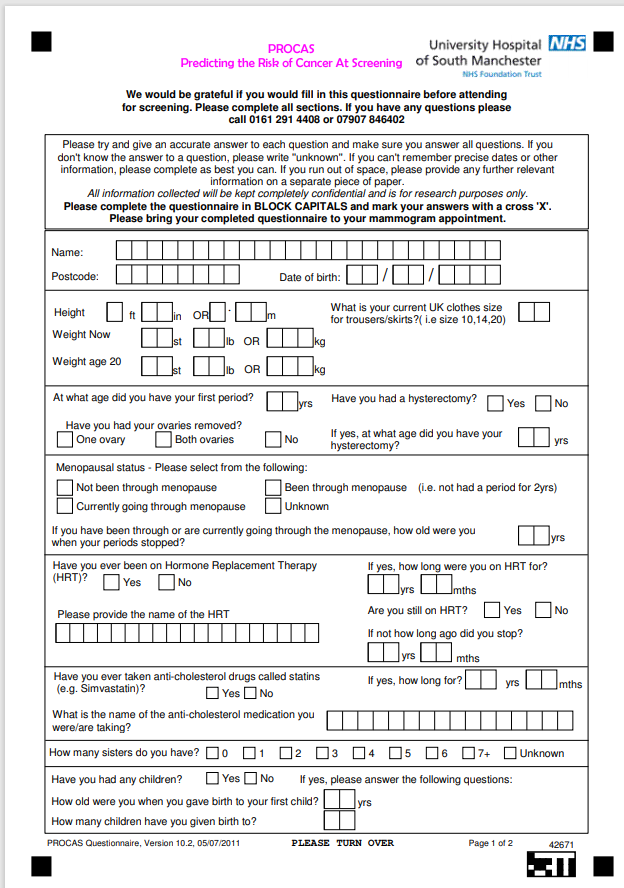

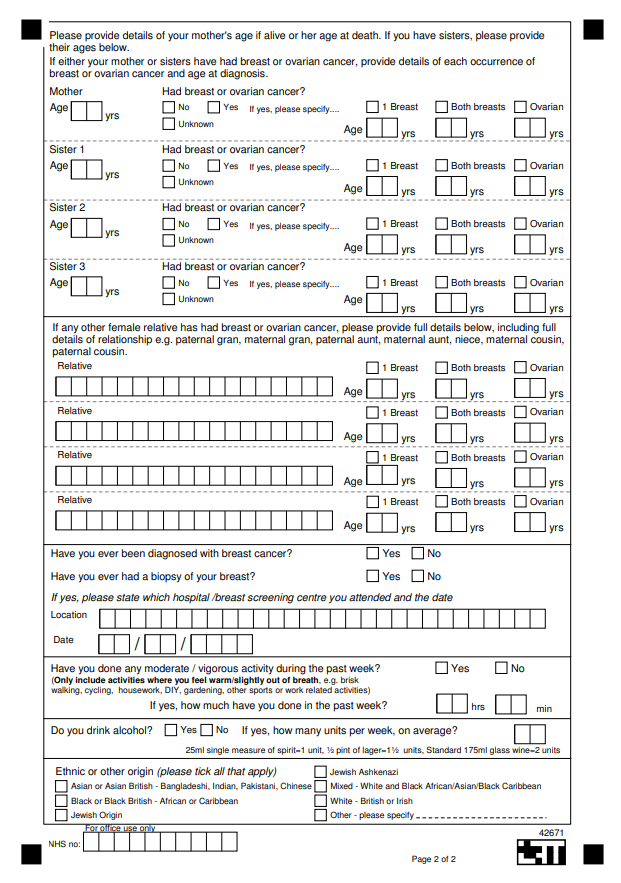
**

Supplement: Supplementary file 1 — Additional file 1. Figure S1: PROCAS questionnaire. [file 12905_2023_2162_MOESM1_ESM.docx]
